# Why is it so hard to improve physicians’ health? A qualitative interview study with senior physicians on mechanisms inherent in professional identity

**DOI:** 10.3205/zma001721

**Published:** 2024-11-15

**Authors:** Heike Schulte, Gabriele Lutz, Claudia Kiessling

**Affiliations:** 1 Witten/Herdecke University, Faculty of Health, Chair for the Education of Personal and Interpersonal Competencies in Health Care, Witten, Germany; 2 Witten/Herdecke University, Faculty of Health, Witten, Germany; 3 Gemeinschaftskrankenhaus Herdecke, Herdecke, Germany

**Keywords:** physicians’ health, resilience, professional identity formation, interviews

## Abstract

**Objectives:**

Current research increasingly describes physicians’ health as endangered. Interventions to improve physicians’ health show inconsistent results. In order to investigate possible causes for weak long-term effects, we examined senior physicians' perceptions about the relevance of their own health and analyzed whether and how these might affect the difficulty to improve physicians' health.

**Method:**

The authors conducted 19 semi-structured interviews with senior physicians from different medical disciplines, analyzed the data and developed theory using the grounded theory method.

**Results:**

Based on the interviews, we developed a conceptual model which identifies reinforcing factors for physicians‘ hesitancy in self-care as well as barriers to change. Participants regarded their own health needs as low and equated health with performance. These perceptions were described as being part of their professional identity and mirrored by the hospital culture they work in. Mechanisms as part of the collective professional identity (CPI) of physicians help to stabilize the status quo through early socialization and pride in exceptional performance. In addition, the tabooing of weakness and illness among colleagues, and dissociation from patients as well as sick doctors were identified as stabilizing mechanisms.

**Conclusion:**

Findings support the assumption that one cause of physicians’ health problems might lie in a CPI that includes tendencies to rate one’s health as secondary or irrelevant. Identified mechanisms against change are, according to Social Identity Theory, typical group strategies which ensure the stability of CPI and make existing attitudes and beliefs difficult to change. However, barriers against change could possibly be overcome by addressing these underlying mechanisms and by a change process that is supported by experienced and competent members of the in-group for the benefit of both physicians and patients.

## Zusammenfassung

**Zielsetzung:**

In der aktuellen Forschung wird die Gesundheit von Ärzt:innen zunehmend als gefährdet beschrieben. Interventionen zur Verbesserung der Gesundheit von Ärzt:innen zeigen uneinheitliche Ergebnisse. Um mögliche Ursachen für schwache Langzeiteffekte zu erforschen, waren die Ziele der Studie, die Wahrnehmung von leitenden Ärzt:innen über die Relevanz ihrer eigenen Gesundheit zu untersuchen und zu analysieren, ob und wie sich diese auf die Schwierigkeit, die Ärztegesundheit zu verbessern, auswirken könnte.

**Methode:**

Es wurden 19 halbstrukturierte Interviews mit leitenden Ärzt:innen verschiedener medizinischer Fachrichtungen durchgeführt und anschließend die Daten mittels Grounded Theory analysiert und zu einem konzeptuellen Modell verdichtet

**Ergebnisse:**

Auf der Grundlage der Interviews wurde ein konzeptuelles Modell entwickelt, das Faktoren für das Zögern der Ärzte bei der Selbstfürsorge sowie Barrieren für Veränderungen identifiziert. Die Teilnehmer:innen schätzten ihre eigenen Gesundheitsbedürfnisse als gering ein und setzten Gesundheit mit Leistung gleich. Diese Wahrnehmungen wurden als Teil ihrer beruflichen Identität beschrieben und spiegeln sich in der Krankenhauskultur wider, in der sie arbeiten. Mechanismen als Teil der kollektiven beruflichen Identität (KPI) von Ärzt:innen tragen dazu bei, den Status quo durch frühe Sozialisation und Stolz auf außergewöhnliche Leistungsfähigkeit zu stabilisieren. Darüber hinaus wurden die Tabuisierung von Schwäche und Krankheit unter Kolleg:innen und die Distanzierung von Patient:innen sowie kranken Ärzt:innen als stabilisierende Mechanismen identifiziert.

**Schlussfolgerung:**

Die Ergebnisse stützen die Annahme, dass eine Ursache für die Gesundheitsprobleme von Ärzt:innen in einer KPI liegen könnte, welche die Tendenz beinhaltet, die eigene Gesundheit als zweitrangig oder irrelevant einzustufen. Bei den identifizierten Mechanismen zum Schutz vor Veränderung handelt es sich nach der Theorie der sozialen Identität um typische Gruppenstrategien, die die Stabilität der KPI gewährleisten und eine Änderung bestehender Einstellungen und Überzeugungen erschweren. Die Hindernisse, die einer Veränderung entgegenstehen, könnten jedoch möglicherweise überwunden werden, wenn diese zugrundeliegenden Mechanismen adressiert werden und der Veränderungsprozess von erfahrenen und kompetenten Mitgliedern der In-Group unterstützt wird, was sowohl den Ärzt*innen als auch den Patient*innen zugutekommen würde.

## 1. Introduction

We have known for some time now that becoming a doctor can be a health risk. Research has shown a decrease in students’ resilience over the time of their professional training [1], [2] and a high prevalence of burnout in medical trainees and junior doctors [3], [4], [5], [6]. Physicians also present several other health issues, including higher rates of depression, anxiety, suicide, stress, emotional exhaustion compared to the overall population [5] as well as increased cardiovascular mortality [7]. Burnout has been associated with reduced quality outcomes for patients and decreased physician productivity [8], [9], [10], which results in considerable costs at a societal level [11], [12].

Over the past years, programs have been implemented to enhance physicians’ health and to reduce burnout and stress, most frequently applying resilience or mindfulness trainings [13], [14], [15], [16], [17], [18], [19], [20]. Resilience in this context refers to the ability to successfully adapt to acute or chronic stress and stay healthy despite negative stressors, such as stressful events [21]. Systematic reviews reveal that the effectiveness of these interventions remains inconsistent, and it is unclear if positive effects are sustainable in the long term [22], [23], [24]. This raises the question of potential barriers to an improvement of physicians’ health behavior which are not yet sufficiently understood but may limit the effectiveness of resilience programs. These barriers may exist on an individual level, on the level of the medical profession as a group, or be based on professional identity and on organizational or systemic factors [7].

We know from the literature that there is inconsistency between verbal attitudes and overt actions on the individual level [25] with a large body of research explaining and further investigating this issue. Little research has been done about the influence of the professional identity on attitudes and actual behavior in relation to health. While the professional identity has been shown to be important for physicians in many respects, there is no generally accepted definition of the concept, and definitions vary greatly [26], [27]. According to Cruess et al. [28], the process of professional identity formation (PIF) is an adaptive developmental process of individuals “organizing their experiences into a meaningful whole that incorporates their personal, private, public, and professional “selves”” ([28], p.2), which is related to three domains influencing and developing identity. The individual domain includes personal characteristics, beliefs about one’s self, and the impact of multiple life experiences. The relational domain expresses the influence on the identity of significant private and professional individuals. The collective domain “reflects the impact of the social groups to which an individual belongs or wishes to join” ([28], p.2). We will call this domain the collective professional identity (CPI). While we do know that attitudes, beliefs, and perceptions change in the PIF process in general [28], [29], [30], [31], little research has been done so far on how the PIF process might shape perceptions and attitudes about physicians’ health.

In this study, we explore perceptions held by physicians about the relevance of their own health and whether these perceptions might influence their willingness to practice self-care. Seeking to understand the sociopsychological processes underlying physicians’ insufficient self-care and the fact that interventions to improve health only show inconsistent evidence, our research questions (RQ) are:

- RQ1 – What kind of perceptions do physicians hold about the relevance of their own health, which might inhibit proper health behavior in physicians?
- RQ2 – Are there factors within the CPI which reinforce these perceptions and prevent change?

## 2. Methods

A qualitative research approach using semi-structured interviews and applying grounded theory methodology was chosen to collect and analyze information on the perspectives of senior physicians and to inductively develop new theory based on the data examined [32], [33][.

### 2.1. Research team

The interprofessional research team contributed pertinent characteristics and reflexivity to the qualitative assessment [34]. The research team consisted of a psychologist with qualitative research experience and a background in leadership development (HS), a physician and head of a psychosomatic department with qualitative research experience in reflective practice formats and PIF (GL), and a physician and professor with experience in qualitative and quantitative research on medical education (CK).

### 2.2. Participants and sampling

We conducted the interviews between January and October 2020. We selected attending and chief physicians as interview partners, because they have successfully established a career in the medical world and will likely have internalized attitudes that are typical of the profession. The study population consisted of physicians from internal medicine, general surgery, and radiology. Fields were selected with the goal of creating a heterogeneous sample in terms of gender, quality, and quantity of interaction with patients and technology as well as the level of invasiveness (cutting and non-cutting) and work setting. 

Recruitment involved email and face-to-face invitations. All interview partners volunteered to participate in the study and did not receive reimbursement. In keeping with the constructivist grounded theory method, we started with easily accessible interview partners and progressed to purposive and then theoretical sampling to the point of theoretical saturation [35].

### 2.3. Data collection

The interview guide was developed by the research team based on the grounded theory approach [32], [33]. HS piloted the interview guide in two interviews and the research team refined the guide afterwards. Experiences gained during the interviews were meticulously analyzed by the research team in regular meetings to remain aware of how the interviewer’s identity and background informed her data collection process [36]. Interviews were conducted by HS in one-on-one settings at a time and place of the participant’s choice. Interviews took 30-93 minutes (average duration 64 minutes), were audio-recorded, transcribed, and pseudonymized. 

### 2.4. Data coding and analysis

We followed the iterative process of data collection, coding, and analysis characteristic [32]. We began our analysis of the transcripts by generating codes to describe and classify the phenomenon under consideration (open coding), followed by an identification of relationships between categories (axial coding), and ending with selective coding to develop a conceptual model (theory) of our research [32]. The researchers HS, GL, CK and a resident with experience in qualitative research in PIF participated in coding and discussing the first ten interviews until an initial conceptual framework was found. We discussed the framework with two external auditors, both physicians and involved in undergraduate medical education, to enhance the trustworthiness and credibility of the emerging theory. Subsequently, HS continued the coding, while the research team met regularly to discuss transcripts and emerging theory by way of inductive-deductive reasoning. This included reflections on how team dynamics and personal attitudes towards health could potentially influence our decision-making process, as well as considerations of methodological and contextual factors. As our conceptual framework advanced, we started reviewing and discussing relevant literature, looking for a theoretical foundation to anchor our central categories and their relationship to each other, a process that was supported by memos and different types of visualization. After the analysis of nineteen interviews, no new significant data appeared, and after another review with the external auditors we closed the data collection process. We used the software MAXQDA 2020 (VERBI Software, Berlin, Germany) for data management and coding. 

### 2.5. Ethical approval 

Authors obtained approval from the Witten/Herdecke University ethics committee (application no. 214/2019) and obtained written informed consent from each participant.

## 3. Results

### 3.1. Characteristics of interview partners

We conducted 19 interviews (15 face to face in presence, one via videoconference, and two via telephone). Of these, six interview partners were females, 13 males. Age ranged from under 40 (one person) to over 60 (two persons), nine were between 41 and 50 years of age, and seven between 51 and 60. They represented different work settings: nine interview partners came from the field of internal medicine, five from surgery and five from radiology. Of these, eight worked in a private, five in public, and six in ecclesiastical hospitals.

### 3.2. Conceptual model

**Figure 1:**
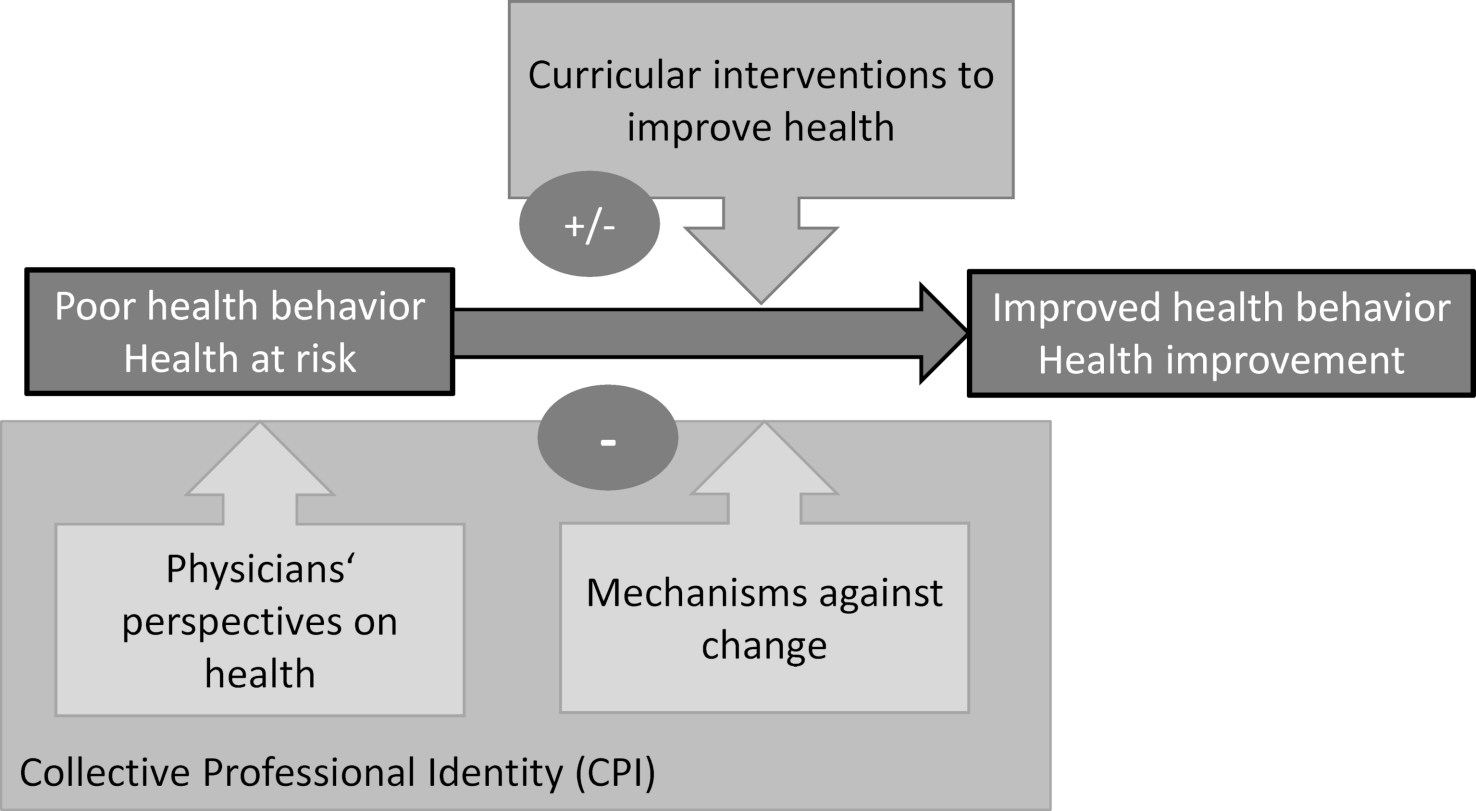
Conceptual model of barriers to the success of interventions to improve physicians’ health behavior

Our conceptual model (see figure 1 (Fig. 1)) explains factors which can prevent the success of interventions aimed at improving physicians’ health behavior. Firstly, physicians perceive their own health needs as unimportant and prioritize their ability to perform and their commitment to service over their health. Secondly, this tendency is rooted in their CPI. Thirdly, they tend to resist change and maintain the status quo. The mechanisms behind their tendency to resist change are also inherent in their professional identity and include early socialization into the physician-in-group, a sense of pride in exceptional performance, and the ability to suppress their own health needs. Additionally, weakness and especially mental illness among physicians are often tabooed, and physicians are inclined to dissociate themselves from patients and sick doctors.

### 3.3. RQ1 – What kind of perceptions do physicians hold about the relevance of their own health, which might inhibit proper health behavior in physicians?

#### 3.3.1. Own health needs are regarded as insignificant

Physicians said they suppress their own health needs and disregard their stress limits. They stated that they simply are not sick and are immune against disease:* “Yes. One is not sick, all right? I've never been sick either, my whole life.”* (G5) This appeared as an irrational and excessive notion of invulnerability which runs through the history of the medical profession. Even the plague doctor *“(…) didn’t get sick from the plague because he was busy nursing the plague patients. He had a certain immunity to the disease.”* (G5) But not only do they perceive a certain immunity. They also seem to not have moral permission to be sick. The high responsibility to the health, even the life of the patient does not allow *“blunders”* (G12) or weaknesses. Own health is regarded as low in the face of this high responsibility for the weak and sick. The physicians most dedicated with *“the best intentions and do their best (…) are most at risk.”* (G14) This conviction even seemed right in hindsight, when this poor health behavior already had led to health damage. The duty to the patient was felt to be much more important than one’s health. It was even mentioned that neglect of one’s own health with resulting *“insomnia”* (G4) and *“hearing loss”* (G4) was the *“toll”* (G4) you had to pay, but that this was nevertheless good and right. 

A few statements revealed that some change might be expected over time. Some of the older physicians stated that younger doctors have more courage to take care of their health and set limits. *“They feel ill more quickly and worry about themselves”* (G4).

#### 3.3.2. Health equals performance and commitment justifies the neglect of personal health needs

Performance and commitment to service for the patient are valued very highly and thus one’s own health is rated as secondary. Interview partners underlined how important it is to be able to work even if one is sick. They recommend taking your attentiveness away from self-awareness and not asking yourself: *“where does it hurt today” (G3). Even basic life needs like being “thirsty”*, being *“hungry”*, *“urgent need to go to the toilet”* (G9) are to be suppressed in favor of the service to the patient. 

This seems to be experienced as something very fulfilling, something *“that’s carrying you”* (G2). Participants described their service to the patient and the fact that one is needed in a social system as something meaningful and as something that makes one healthy: *“But basically, work and being needed and being involved in a social system are part of being healthy and happy”.* (G5)

#### 3.3.3. The low importance of personal health is mirrored by the system, i.e. the hospital culture

These perceptions seem to reflect not only an individual perspective. Performance and disregard for their own health seem not only to be experienced by single doctors, but to be expected of physicians on a systems level. There seems to be little attention to employee health needs in organizations, physicians don’t feel cared for by their employers, but simply must perform as *“passive cogs in the wheel”* (G2). One participant mentioned: *“It doesn't matter at all. Nobody here cares. Whether we are healthy or not healthy.”* (G12) Or as another interview partner put it: *“Yes. Nobody cares. The store must run.”* (G4) So, on the organizational level again, the functioning of the system is prioritized higher than the individual’s health. 

### 3.4. RQ2 – Are there factors within the CPI which reinforce these perceptions and prevent change?

While analyzing the collated data, we identified specific indicators that these perceptions are ingrained in physicians’ CPI. When questioned about their attitudes, interview partners tended to generalize and talk about physicians as a group, referring to themselves as “one”, “we” and “us”, like this physician who summarized her attitude regarding the relevance of her own health as follows: *“We must function. When we are sick, we’ll be mocked.”* (G12) Furthermore, we encountered the above-described perceptions in all interviews, which indicates that they are part of a shared, collective belief system. These indicators support the impression that it is part of physicians’ CPI to set aside their own needs, to ignore and neglect signs of weakness or illness, and to view their own health as irrelevant, as long as they are able to perform. 

Part of the CPI was not only this tendency to disregard self-care in favor of performance, but participants also described mechanisms which seem to ensure that the reported CPI remains intact, and to protect the status quo against change. 

#### 3.4.1. Early socialization

The tendency to disregard one’s own health starts early in the medical career and is passed on through medical education and residency. There seems to be high expectation pressure from early on not to be sick and not to be absent. Physicians describe almost a given law not to be violated in their residency to come in even if one is sick. A chief physician illustrated this in the following statement: *“Well, I was socialized in such a way that one wasn’t ill at all. One wasn’t absent from work.”* (G5) In connection with this pressure many interview partners report high levels of distress, and pressure even to the point that they* “decompensated and left (…) because they just couldn’t take it anymore.” *(G18). 

If they succeeded in carrying through and stayed in the job, they expressed the notion that if they had to endure it, others should also endure it in order to join the group or, as one chief physician put it: *“It’s like celibacy. I had to endure that too, so I don’t want to abolish it now.”* (G7). 

#### 3.4.2. Pride in exceptional performance

Our interview partners reported that by neglecting stress limits they discovered unsuspected capacities. One physician reported *“Because we know that when you need to, you can always handle more. You can go on even when you think you can’t take it anymore.”* (G9). For many, this exceptionally high level of resilience and performance is associated with positive emotions, notably pride. Belonging to the group of physicians and being able to meet these high demands is an exceptional privilege and also distinguishes oneself from others as especially strong: *“Not everyone can pull this off, that's for sure.” *(G3) The ability to do so generates positive emotions. *“So that's the big attraction. That's the great stress in surgery, too, of course. Phases, of course, where you have to work at an incredibly high stress level.”* (G11) Surgeons expressed this pride most strongly: *“So being sick is unsurgical. That's always been our saying, which definitely exists in surgery.”* (G5)

#### 3.4.3. Weakness and illness are taboo

In many interviews physicians experienced their weakness or limits as something that is a failure: *“I think admitting weaknesses, saying I can’t do it or I don’t want to do it, is also a failure in a way.” *(G18) Not only expressing weakness, but even thinking about it is impossible:* “One should even become resistant to unfavorable thoughts (…) mustn't think about it too much (…) I can't think about it every day.”* (G3). It was also seen as not only a personal failure, but a taboo, that means prohibited or restricted by social custom, by the medical community. In this context, several topics were regarded as taboos: weakness, burnout, depression, suicidality, defeat, needs, and substance addiction. Breaking the taboo seemed to impede career development, as one interview partner stated:* “That is of course something that is still a bit taboo, weakness.”* (G11) Especially mental illness and reference to the high suicide rates among medical professionals are considered a taboo.* “But you’re also afraid of this. (…) What kind of look do you think the others would give me.”* (G12) This taboo goes as far as complete suppression of memories about colleagues who committed suicide, which is evident in the following statement: *“I can’t think of anyone (…) who’s killed themselves. [… thinks…] Yes, there’s one. (…) a colleague in anesthesiology (…) And the other day in the hospital next door, I think an anesthetist took his own life. And (…) I think we had three suicides here (…) in the clinics.” *(G8)

#### 3.4.4. Dissociation from the inefficient and the sick 

Another mechanism to protect against change described was dissociation from the sick. In order to protect the image of physicians as high performers, they tend to distance themselves from sick colleagues. They are not described empathically, but rather as *“just not good enough”* (G2) and that they don’t belong to the team anymore. A doctor even described her relief after a colleague suffering from addiction left the clinic: *“And when he left the clinic, I was happy in a way, like “look, we don’t have him in the clinic anymore””* (G12).

The need to block out illness for one’s own professional group also becomes evident in dissociation from the sick patients. A clear boundary is drawn between patients and doctors. *“The word is not actually used, namely physicians’ health. There are the patients. And there are the non-patients. The staff, so to speak.”* (G6) Another doctor explained it like this: *“Because you then (...) distance yourself from the sick and the misery that lurks behind it and the suffering that can lie behind it, because you want to distance yourself clearly from it.”* (G14) They described the reason for this distancing mechanism as self-protection: *“You see so many illnesses where you simply say, no, this affects others and I am the helper and these are the sick. And that you somehow don't want to admit it at all in the context of self-protection, because you see what horrors can befall you from one moment to the next.” *(G14) 

Further illustrative quotes are summarized in attachment 1 .

## 4. Discussion

Effects of initiatives to improve physicians’ health have been reported as being inconsistent and the sustainability of positive effects seems unclear [22], [23], [24]. In this study, we set out to better understand why it is so difficult to improve physicians’ health, with the focus on physicians’ perspectives on their own health as a potential root cause of the problem. The results of our study show specific perspectives on physicians’ health and how they are deeply engrained in their professional identity. They also reveal mechanisms which prevent change of this part of the identity.

Based on our findings, we were able to demonstrate that physicians tend to delay self-care and show deep-seated values of prioritizing work, which is supported by a large body of evidence published in recent years [7], [37]. These perspectives were embedded in a collective professional identity as these perspectives seem to be shared by the collective of our interview partners, regardless of gender or medical discipline. Several authors have described these phenomena as being part of a specific culture of the medical profession [7] to ignore weakness and illness and to exhibit a strong sense of duty to the point of total exhaustion [38], [39], [40]. It seems like there is a hidden curriculum pertaining to the irrelevance of physicians’ own health. Beresin et al. summarize the hidden curriculum regarding physicians’ own wellbeing as “the reluctance to admit weakness, expose our shame of suffering from the stigma of a psychiatric disorder, or even discuss the pressures we share” ([5], p.9). Many publications ascertain a detrimental influence of this hidden curriculum on physicians’ health [41], [42], [43], [44]. Our data support Beresin’s definition but also point beyond it. Our findings indicate that in addition to the social dimension Beresin et al. refer to, it seems to be part of physicians’ CPI to ignore and deny their own weakness and illness.

In addition, we were able to reveal mechanisms as part of physicians’ CPI which prevent physicians from setting their health as a higher priority, which are early socialization, pride in exceptional performance, tabooing weakness and illness, and dissociation from the inefficient and the sick. As these mechanisms in combination with CPI were conspicuous and might add new insight to the existing evidence, we discuss them in more detail. 

Our interview partners distinguished between their in-group and two out-groups – the group of patients and the group of sick physicians. It has been frequently reported how challenging it is for unwell physicians to step into, accept, and identify with, the patient role [45]. This underlines how difficult it is for physicians to handle their group status flexibly and how personal health issues are ignored to ensure in-group-membership. Our findings correspond with Tajfel and Turner's social identity theory (SIT) [46], which shows how the mechanisms we identified are typical process factors in groups to ensure conformity of group members and stability of CPI [46], making it difficult and risky for physicians to notice and address their own weakness or illness. The SIT describes that groups differentiate between in-group and out-groups and that the desire to stay in the in-group is a typical mechanism in groups [46], [47], [48]. 

Another mechanism we identified is pride in the ability to deliver exceptionally high performance and even cross boundaries. Pride strengthens the CPI [49], [50], but positive emotions also reinforce the adoption of the CPI as part of the individual self-concept [46]. We know that individual and social identity in medicine are closely intertwined [51], [52]. This is critical because unwell physicians risk more than merely loss of the in-group membership. If they are unable to maintain their high performance, they will also be deeply unsettled in their individual identity [53] and risk failure in their career. 

An additional mechanism according to our findings is the early socialization into the established attitude and belief system. This early socialization process has been reported in other studies, where medical students described the “pressure to prove themselves worthy of the profession” ([54], p.131) and the implicit and explicit message that failure, weakness, or inadequate emotional control lead to exclusion from the system as a constant experience from day one [54], [55]. This early and consistent message pertaining to what the SIT calls the prototype of a good group member is essential to maintain a strong group identity [45], [56], as only those who share the collective belief system remain in the group.

This socialization process is reinforced by taboos regarding one’s own weakness, illness, and high suicide rates [7]. Taboos are an important mechanism for securing collective identities, and group membership implies acceptance of the taboos associated with the CPI [57], [58]. Our findings are supported by studies showing that the emotional challenges of becoming a physician are often a taboo topic in medical training [55] that can lead to a *“culture of silence”* ([53], p.1) about emotional and mental health struggles associated with the frequency of physician suicide [53].

These findings help us understand why it might be so hard to improve physicians’ health. It seems to be deeply ingrained in the CPI of physicians to view their own health as irrelevant and to ignore personal needs and weaknesses. This is a cause for serious concern, as professional values and norms impact on a fundamental level how we behave, learn and change [25], [59], [60]; moreover, values and norms which are part of a CPI, are particularly difficult to change [46]. 

### Limitations 

In this study, we relied on the spoken word of physicians rather than an objective measurement of attitudes. Consequently, this study does not quantify attitudes, but merely provides a qualitative understanding of physicians’ attitudes and perceptions. As a qualitative study, only a small group of physicians were included, all willing to participate in the study. We do not know whether a study with a different group of physicians’ might have come to similar or other results. Generalizability of findings needs to be evaluated in further studies. It cannot provide clarity on causes and effects, either. Therefore, while this study might provide a foundation, more research needs to be done to better understand and potentially develop measurement tools for attitudes and perceptions around physicians’ own health. We performed the research in Germany, and the local (medical) culture necessarily informs our results. We also interviewed senior physicians in hospital settings only. However, in training, every physician spends years working in hospitals. Therefore, hospitals serve as places of systemic socialization. Still, further research will be interesting that elaborates on and refines our theory in other work settings, other countries, and other medical fields. Lastly, even though we carefully monitored the process, the interviewer’s background as a psychologist and representative of an external group might have affected the content provided by the interview partners.

## 5. Conclusions

The implementation of trainings to improve physicians’ health without addressing the underlying mechanisms becomes questionable in light of our results. To sustainably improve physicians’ health, we need a change in the perception and value of physicians’ health that is part of physicians’ CPI and that is conveyed and protected by mechanisms woven into everyday experience from day one of medical school. This is a major task, and it needs to be led from the inside. Attempts to change attitudes in groups have shown greater impact where the change was initiated and supported by members of the in-group [61], [62] who were perceived as credible, i.e. experienced and competent [63] and who were prototypical group members [64]. Therefore, we need experienced, successful, and credible physicians who question the old ways, teach about the impact of physicians’ health on the quality of care, address the mechanisms within the professional identity, function as role models, and lead this change. It might be worthwhile and a starting point for a necessary shift. 

Our results provide an additional perspective on the root causes of our struggle to improve physicians’ health and an opportunity to invite transformation to physicians’ CPI that values not only their patients’ health but also their own.

## Authors’ ORCIDs

- Heike Schulte: [0009-0001-6706-1815]
- Gabriele Lutz: [0000-0001-5044-8485]
- Claudia Kiessling: [0000-0003-4104-4854]

## Acknowledgements

The authors wish to thank the interview partners who took the time to participate in this study. Our thanks go to Christina Wagner, interpreter and translator, for linguistic assistance, Cornelia Preusse, Clarissa Frehle, Katharina Knie, Florian Mennigen, and to the IAP Team at the Witten/Herdecke University for support. 

## Competing interests

The authors declare that they have no competing interests. 

## Supplementary Material

Explanatory quotations of interview partners to illustrate the concept model and categories
