# Supplementary material for: Why is it so hard to improve physicians’ health? A qualitative interview study with senior physicians on mechanisms inherent in professional identity
Source: GMS J Med Educ. 2024 Nov 15;41(5):Doc66. doi: 10.3205/zma001721 (PMC11656177; doi:10.3205/zma001721)
Supplement: Explanatory quotations of interview partners to illustrate the concept model and categories [file JME-41-66-s-001.pdf]

Attachment 1: Explanatory quotations of interview partners to illustrate the concept model and categories

**RQ1 – What kind of individual perceptions do physicians hold about the relevance of their own health?**	
I=Interviewer; G=Interview partner	
**Category**	**Explanatory quotations**
Own health needs are regarded as insignificant	"Yes. One is not sick, all right? I've never been sick either, my whole life. (…) It's a bit like the old doctors, the plague doctor, who didn't get sick from the plague because he was busy nursing the plague patients. He had a certain immunity to the disease." (G5)<br>"We don't ask for help. This is something we can handle by ourselves. We are not sick. We can cope with all that." (G12)<br>"Us doctors (…) must always be strong and do a good job, be confident, because we have an immensely responsible job. We can't afford blunders." (G12)<br>"I'm not healthy. Clearly, 40 years of night duty have taken their toll. I have insomnia, I have hearing loss, uh, and although I have tried to distance myself. It still took its toll (…) I think I've done quite a lot right in my professional life." (G4)<br>"Or well, then I just take some sleeping pills and get back into my rhythm, because the biorhythm is one of the first things to go, apart from losing half of your friends when you're in hospital all the time. And I think that's where the danger lies, precisely because it remains unreflected. That it is actually the people who have the best intentions and do their best that are most at risk." (G14)<br>"There has also been a social change at this point. The right to illness, for example, is a development of the last 10-15 years" (G5).<br>"So I think that younger colleagues today are already paying more attention to themselves. They feel ill more quickly and worry about themselves. And they are also quicker to take a break for 1, 2, 3 days than the old generation did. That is absolutely certain." (G3)
Health equals performance and commitment justifies the neglect of own health needs	"You have to be resilient. You can't ask yourself every day 'where does it hurt today' (…). That doesn't work. Just get going. Do." (G3)<br>"Because to be honest, we are used to continuing with our work even though we are tired. Even though you're thirsty. You're hungry, you urgently need to go to the toilet. You can pull yourself together if you know that this must be done now." (G9)<br>"I dragged myself to the clinic with 38.5 degrees fever. There was simply no such thing as being sick." (G5).<br>"I don't judge myself too harshly because I know I have to get through the next day and the next day and the day after that and the next weeks and months and years. (...) To be ready. To stay fit for work. Yes. Sure." (G3)<br>"And most of the colleagues I know, they can't go home unless all the work is done. (...) I have to have seen that at least once, even if it's long after work. I can't go home without that, because I also have a responsibility. I can't say it is 4 p.m. now, so it doesn't matter." (G4)
The low importance of own health is mirrored by the system, i.e. hospital culture	"It doesn't matter at all. Nobody here cares. Whether we are healthy or not healthy." (G12)<br>"And we are expected to be strong. We have to be strong. We have to help." (G4)<br>I: "Which role does physicians' health play in the clinic (...)?"<br>G10: "None at all."<br>G9: "That is true. None at all. Exactly. Nobody pays any attention to that."<br>"It was actually the case in our department that certain things that actually contribute to health were not taken into account. So our computer program for the duty roster regularly sounded the alarm because time limits according to health and safety regulations had already been exceeded." (G18)<br>"These are vicious circles that I think begin to take on a life of their own. That you have more and more work and more and more work and ultimately the mountain that lies behind you and is not yet finished becomes bigger and bigger and on the other hand it is always demonstrated to you, or the others don't say anything and just act as if they can do everything. (...) The self-experience and the experience of the others or the experience of the environment and that you are ultimately also compared or ultimately presented with examples of comparison that are either irrational or far removed from reality." (G17)

RQ2 – How are these individual perceptions of physicians reinforced by the collective professional identity formation?	
**Category**	**Explanatory quotations**
Early socialization	"Even in medical school, there were so many of us, and it was clear that individual students were not important. You were a number and had to go your own way. In the profession, the unspoken message was that there were 100 others. If you don't want to do it, we'll find someone else." (G9)<br>"In my first year, I used to think that it wouldn't really matter if I got hit by a truck now (…) there were people who simply decompensated and left (...) because they just couldn't take it anymore." (G18)<br>"As a resident, one would also come in sick, no matter what." (G18)<br>"We started with a very direct, aloof, choleric boss. I wouldn't have dared to ask him for help. Because I knew he would have interpreted that as a sign of weakness. And he wasn't looking for weak employees. That was at the very beginning of your professional life, when you are still not self-assured. Where you don't know if you'll be employed here. I had a temporary contract. And that was also to be extended." (G1)<br>"I think a major reason is that our employers or supervisors just didn't allow us to do that and we didn't really have a choice. Either you stay in the system and go along with it or you're out." (G6)
Pride in exceptional performance	"The advice to students is, as I said, sincerity, the effort culture that you have to grit your teeth sometimes. (...) And in the knowledge that effort will lead to success at some point. So the effort also brings a feeling of happiness." (G5)<br>"Of course, there have always been views that surgeons have perhaps always seen themselves as heroes and the fathers of medicine in general. And that is still a bit more likely in surgery, also in people's minds. And so somehow their own health took a back seat. Because taking care of oneself was probably not surgical. Because heroes don't pay attention to their own health. They push their limits and save people's lives. And I think that's what they call 24/7." (G11)
Weakness and mental illness are taboo	"And yes, of course, it's still a kind of taboo subject. Addiction in particular. You don't talk about it among colleagues. If anyone does, it's behind closed doors. (...) You don't want people to talk about you like that. You don't really talk about others like that, either. You think it's a kind of weakness. They are simply not fit, not strong enough. Something like that. Well, and they have failed, in a way." (G1)<br>"Yes, and just this basic thinking 'it's always gone well', 'you've never really been ill', and 'you don't really have anything, what's there to worry about now'. But you mustn't think about it too much. You just do your job, don't you? I can't think about it every day." (G3)<br>"There is a department here in the house, there was a doctor, I mean she was also in the psychiatric ward for four weeks recently, because of burn-out. I am in contact with the department occasionally, I didn't notice that before. She just suddenly left, she's been gone for six weeks now, and I don't think she had confided in anyone here, and the structures in her team didn't fit in with her needs." (G15)<br>"Doctors are all healthy, all the way down the line, and I'm probably healthy too. I would also say I'm healthy, but whether that's really the case is another matter. So we don't like to talk about it." (G16)
Dissociation from inefficient and sick doctors	"If you don't make it, you're out of the running. You have to get through if you want to be a doctor. You have to (...) If you don't make it, you're just not good enough." (G2)<br>"Yes, he is not one of us. We are not sick. We are not people addicted to pills. We are strong. We don't go into burnout. We are very resilient, we are a pillar of society. We are needed. We must not show any weakness. It doesn't work that way, of course it doesn't. We are just like all other people, aren't we?" I: "Hmhm. Yes. But it's a bit part of the image. Of the identity?" G: "Yes. Yes. Definitely. Exactly. Yes." (G12)<br>"I once heard about a colleague, here in this clinic, who was probably addicted to pills and also stole things here. So I distanced myself from him inwardly. So in retrospect I feel really bad. I think I've always given him strange looks, like thinking 'Oh my goodness'. But you're also afraid of that. If I look at him like that, how do you think the others would look at me? That would be exactly the same." (G12)<br>"Addiction in particular. You don't talk about it like that among your colleagues. If they do, it's behind closed doors (...) They don't really talk about others that way either. They think it's weakness somehow. They are simply not capable, not strong enough. Something like that. Yes, and then he has somehow failed." (G1)

Dissociation from patients	“Okay, I'm not going to shuffle around the ward with my hand pressed to my back like one of my patients and tell everyone I have a backache; rather, I'm going to stand up straight in my white coat and put up a brave front. I’ll just take 2,000 mg of ibuprofen a day, if I have to. (...) In order to distance myself from the feeling of being a patient. You see so much illness where you simply say, ‘No, this affects others, I am the helper and they are the sick’”. (G14)<br>“Because it is a reversal of roles. Normally patients come to me because they need help. And if I want help, I would have to take on the other role. I: “Hmhm. And that would be unfamiliar?” G: “It is unfamiliar.” (G4)
